# *Staphylococcus aureus* ZigA is implicated in survival in zinc-deplete and genotoxic environments

**DOI:** 10.1128/spectrum.00454-26

**Published:** 2026-06-11

**Authors:** Kyle T. Enriquez, Andy Weiss, Yasiru R. Perera, Indu Bhatia, Melumo Togashi, Tae Akizuki, W. Hayes McDonald, Walter J. Chazin, Eric P. Skaar

**Affiliations:** 1 Vanderbilt University Medical Scientist Training Program, Vanderbilt University School of Medicine 12327 , Nashville, Tennessee, USA; 2 Department of Pathology, Microbiology, and Immunology, Vanderbilt University Medical Center 204907, Nashville, Tennessee, USA; 3 Vanderbilt Institute for Infection, Immunology, and Inflammation, Vanderbilt University Medical Center, Nashville, Tennessee, USA; 4 Department of Biochemistry, Vanderbilt University 215875, Nashville, Tennessee, USA; 5 Center for Structural Biology, Vanderbilt University 685053, Nashville, Tennessee, USA; 6 Mass Spectrometry Research Center, Vanderbilt University 5718, Nashville, Tennessee, USA; University of Florida College of Dentistry, Gainesville, Florida, USA

**Keywords:** *Staphylococcus aureus*, COG0523 proteins, zinc, nutritional immunity, zinc-binding proteins

## Abstract

Zinc is a trace nutrient transition metal important to the host-microbe interface, as zinc is a cofactor for upwards of 10% of the pan-proteome. Given the importance of first-row transition metals, including zinc, to cellular life, it is predicted that *Staphylococcus aureus* and other pathogens have developed methods to distribute intracellular zinc across a hierarchy of protein clients. COG0523 proteins are a family of GTPase proteins present in all domains of life that act as post-translational regulators of client activity through direct protein-protein interactions. The pathogen *S. aureus* maintains three predicted COG0523 proteins, including ZigA. We confirm that *S. aureus zigA* is regulated by the zinc-responsive transcriptional regulator Zur. We further demonstrate that ZigA exhibits zinc-dependent GTPase activity and, in the presence of GTP, ZigA dimerizes. Co-immunoprecipitation and yeast-2-hybrid approaches resulted in a prioritized list of potential interacting partners for ZigA, including proteins involved in DNA repair, and in protection against oxidative stress. The loss of ZigA resulted in modest growth and survival defects when *S. aureus* experiences low zinc and DNA-damaging stresses, including UV crosslinking and reactive oxygen species. The loss of ZigA was not associated with phenotypes in the setting of other stressors, including iron limitation, antibiotic use, or stressors associated with COG0523 activity in other bacteria. During systemic infection, the loss of ZigA appears to only marginally impact *S. aureus* pathogenesis in the lung. Taken together, these data suggest that Zur-regulated ZigA may act as a dimer to support zinc-dependent, DNA damage repair by multiple mechanisms.

**IMPORTANCE:**

In this work, Enriquez et al. study the response of *Staphylococcus aureus* to zinc limitation. Among these genes are those encoding the COG0523 proteins, which in bacteria and eukaryotes can act as metallochaperones. Zinc plays critical roles in *S. aureus* DNA damage repair machinery, which is particularly important during infection. This work proposes COG0523 protein ZigA as a minor contributor to staphylococcal pathogenesis in zinc-limited, genotoxic environments. Biochemical approaches suggest that ZigA dimerizes in the presence of GTP and that GTP activity is accelerated in the presence of zinc. Furthermore, the loss of ZigA results in modest *S. aureus* growth and survival defects associated with low-zinc and DNA-damaging environments. Taken together, these data suggest that Zur-regulated ZigA may act as a dimer to support zinc-dependent, DNA damage repair by multiple mechanisms.

## INTRODUCTION

*Staphylococcus aureus* is a leading cause of infectious morbidity and mortality across the globe, contributing to more than a million deaths annually (1–3). *S. aureus* pathogenesis is dependent on the capture and utilization of nutrient transition metals, including, but not limited to, zinc (Zn^2+^) (4–10). In environments depleted of transition metals, transcriptional factors, including Zur, Fur, and MntR, release bound metal ions, which decreases their affinity for DNA-binding sites. As a result, transcript abundance of their respective regulons is increased to respond to the stress associated with low transition metal abundance (5, 7, 8, 11). The ability to respond to altered metal concentrations is fundamental to microbial pathogenesis, and, in turn, innate immune cells produce proteins that sequester transition metals at the host-microbe interface (10, 12–15). Collectively, these strategies, wherein nutrient metals are used by the host to limit pathogenesis, are termed nutritional immunity (6, 8–10). Nutritional immunity is a highly effective strategy for preventing bacterial growth, as Zn^2+^ alone is predicted to participate in the structure and thereby function of 5%–8% of bacterial proteins (16–19). The high biological demand for Zn^2+^ is juxtaposed with the scarcity of free Zn^2+^, on the order of pico- to femto-molar concentrations within biological tissues (8, 16, 20–23). This requirement for Zn^2+^, combined with the existence of multiple metal-sensitive transcription factors, suggests that pathogens use discrete systems to allocate Zn^2+^ and other trace nutrient transition metals to metalloprotein clients in response to diverse stressors experienced at the host-microbe interface.

COG0523 proteins are highly conserved members of the G3E P-loop GTPase family of proteins that contain metal-binding (CXCC) and Walker A and B NTPase domains (4, 24). These domains are key to the enzymatic activity of COG0523 sub-family members, which have been characterized in bacteria and eukaryotes as transition metal-dependent regulatory proteins (25–31). COG0523 proteins have been demonstrated as metallochaperones of nickel, cobalt, and zinc ions, suggesting a conserved function of these metal-binding GTPases (4, 26, 29, 31–35).

Members of the COG0523 protein family have been identified in eukaryota, *Helicobacter pylori*, *Acinetobacter baumannii*, *Bacillus subtilis*, and other bacteria (26, 28–31, 33, 36). Phylogenetic analysis of COG0523 proteins consistently identifies a core five groupings within this protein family, including those implicated in synthesizing endogenous vitamin B12 (CobW-like), activating NHases (UreG-like), responding to carbon starvation (YjiA-like), and overcoming Zn^2+^-limitation (YeiR-like and YciC-like) (4, 24). For instance, eukaryotic ZNG1 is critical to proteostasis through the transfer of Zn^2+^ to activate METAP1, which has significant consequences for cellular homeostasis in *Homo sapiens*, *Mus musculus*, *Danio rerio*, and *Arabadopsis* species (4, 28, 30, 31). In bacteria, YeiR/YciC-like COG0523 sub-family members aid pathogens’ responses to Zn^2+^ limitation in combination with other cellular stressors. *A. baumannii* MigC supports resistance to cell wall stress in a Zn^2+^-dependent manner by interacting with the cell wall synthesis protein MurD (36). In *Bacillus subtilis*, YciC was re-annotated to ZagA (ZTP-activated GTPase A) after it was discovered that *zagA* abundance increases in low Zn^2+^ conditions and Zn^2+^-bound ZagA interacts with *B. subtilis* FolE, helping maintain genomic integrity in response to folate stress (26). While *S. aureus* ZigA is predicted to belong to the YciC/YeiR sub-families of COG0523 proteins, significant questions remain surrounding the contribution of this protein to pathogenesis in the setting of Zn^2+^ limitation.

Multiple studies have suggested that *S. aureus zigA* (zinc-inducible GTPase A; *NWMN_RS02350*; *NWMN_0417*) is regulated by Zn^2+^ and the Zn^2+^-responsive transcriptional regulator Zur (4, 5, 25, 27). Exposure to the host metal-chelating protein calprotectin, which sequesters Zn^2+^ at sites of inflammation, also results in increased *zigA* expression *in vitro* (5). *S. aureus* ZigA is predicted to be a YciC homolog, supporting a potential role for ZigA in the response to low Zn^2+^ environments (4). *S. aureus* ZigA is a Walker GTPase with Zn^2+^ binding at a high-affinity CICC site and two additional low-affinity Zn^2+^-binding sites (27). Taken together, these data led to the hypothesis that *S. aureus* ZigA contributes to *S. aureus* Zn^2+^ homeostasis and pathogenesis by interacting with yet an unidentified Zn^2+^-binding protein client.

This report contains biochemical and microbiological studies aimed at determining the role of *S. aureus* ZigA in pathogenesis. At a transcriptional and translational level, *S. aureus zigA* is confirmed to be Zur regulated. In the presence of GTP, ZigA dimerizes *in vitro*, and the GTPase activity of *S. aureus* ZigA is also confirmed to be Zn^2+^-dependent. A prioritized list of putative interacting partners derived from co-immunoprecipitation (co-IP) and yeast-2-hybrid (Y2H) experiments led to the investigation of the functional relevance of a potential interaction between ZigA and UvrA. Although we were unable to confirm a functional interacting partner of ZigA, we did observe that loss of *zigA* or mutations of the ZigA CICC/Walker domains resulted in small growth and survival defects in DNA-damaging, low Zn^2+^ environments. These differences are not observed with the depletion of other transition metals and are not present in Zn^2+^-deplete cultures treated with antibiotics. Notably, these defects were observed despite ZigA abundance not changing upon exposure to DNA-damaging agents. Finally, the loss of *zigA* is associated with only a modest defect in CFU obtained from the lung within systemic models of infection. Taken together, these studies demonstrate that ZigA is a Zn^2+^-binding COG0523 protein that dimerizes in the presence of GTP, but do not reveal an environment where ZigA is indispensable to *S. aureus* growth and survival, nor do they identify a high-affinity protein client of ZigA.

## RESULTS

### *Staphylococcus aureus* ZigA exhibits Zn^2+^-dependent GTPase activity as a homodimer

**Fig 1:**
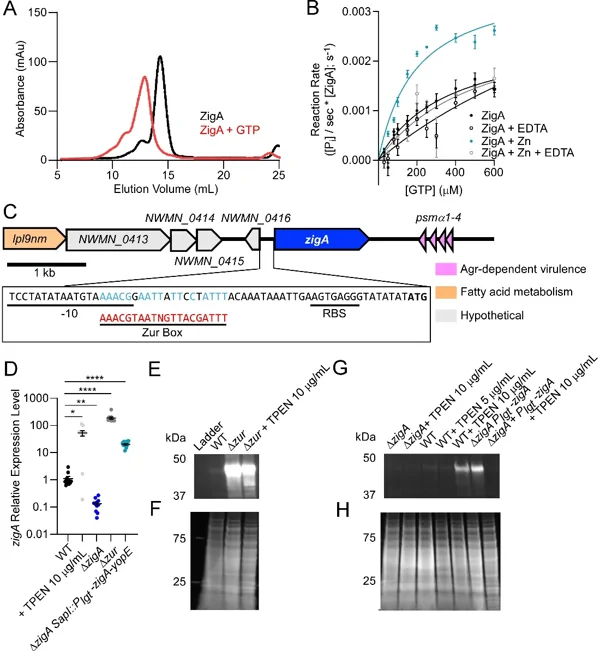
*S. aureus zigA* is regulated by Zn^2+^ and Zur. (**A**) Size exclusion chromatography of purified ZigA monomer with (red) and without (black) added GTP. (**B**) Malachite Green assays of ZigA intrinsic GTPase activity in the absence or presence of Zn^2+^ or Zn^2+^ plus EDTA. (**C**) Genomic context of *zigA* (blue), highlighting the known and hypothetical functions of surrounding *S. aureus* genes, including the intergenic region preceding *zigA*, which contains a sequence (cyan) resembling a canonical gram-positive bacterial Zur box (red). (**D**) Relative expression level (2^−∆∆Ct^) by SYBR Green-based qRT-PCR of *zigA* one hr after treatment of *S. aureus*. (**E and G**) After growth for 24 h in tryptic soy broth (TSB) with or without Zn^2+^ chelator TPEN, the abundance of ZigA (~45 kDa) was measured via immunoblot using rabbit polyclonal α-ZigA 1:5,000 in 1× TBST blocking buffer with 5% (wt/vol) EasyBlocker. Secondary antibody staining was completed using goat α-rabbit Ig 1:10k, then imaged on a Bio-Rad Chemi-Doc. (**F and H**) A parallel gel was used for Sypro Ruby staining as a measure of loading control. Statistics were completed using Brown-Forsythe and Welch’s analysis of variance tests with post-test via Dunnett’s T3 multiple comparison test, with individual variance computed for each comparison to NWMN alone. *P < 0.05, **P < 0.01, and ****P < 0.001.

COG0523 protein function is associated with metal ion binding, GTP hydrolysis, and post-translational regulation of protein client(s) (27, 28, 31, 37, 38); loss of the GTP-binding and/or CXCC Zn^2+^ binding via mutation reduces COG0523 enzymatic activity (24, 33, 36, 39–41). Both zinc binding and NTPase activity have been reported for *S. aureus* ZigA (4, 27, 37). To define the conditions under which *S. aureus* ZigA exhibits GTPase activity, ZigA was expressed and purified for GTPase assays. During purification of the protein, two peaks appeared in the elution from a size exclusion chromatography column corresponding to monomeric and dimeric states of the protein (Fig. S1). In a previous study, *S. aureus* ZigA eluted as a single broad peak in SEC (27). In an attempt to rationalize this difference, we tested adjusting the concentration of ZigA, identity of reducing agent, flow rate, and temperature, but we were unable to reproduce this observation. We will continue to explore the equilibrium between monomeric and dimeric states and will report on our findings in due course. Importantly, in the presence of GTP, this equilibrium shifts drastically from the monomer to the dimer (Fig. 1A), implying the dimer is the functionally relevant state of *S. aureus* ZigA. To further characterize the biochemical activity of ZigA in this functionally relevant state, GTPase activity of ZigA was measured using a malachite green assay. Freshly purified ZigA without added Zn^2+^ (Fig. 1B) exhibits low GTPase activity (*V*_max_ = 2.6 × 10^−3^ s^−1^ ± 0.9 × 10^−3^ s^−1^, *K*_*m*_ = 3.5 × 10^2^ µM ± 2.4 × 10^2^ µM; mean ± 95% CI). Addition of ZnCl_2_ results in a significant increase in GTPase activity (*V*_max_ = 3.6 × 10^−3^ s^−1^ ± 0.4 × 10^−3^ s^−1^, *K*_*m*_ = 1.9 × 10^2^ µM ± 5 × 10^1^ µM; mean ± 95% CI), which is subsequently ablated upon addition of EDTA (Fig. 1B). These results confirm and refine previous characterizations of *S. aureus* ZigA enzymatic activity (27).

### *S. aureus* zigA transcript abundance is regulated by Zur and inversely correlated with environmental Zn^2+^

The genomic context of *zigA* (*NWMN_0417*; *NWMN_RS02350*) was used to develop hypotheses regarding *zigA* transcriptional regulation (42). Upstream of *zigA* and its predicted Ribosomal Binding Site (RBS) is a region that resembles a Zur box (red; Fig. 1C), consistent with literature suggesting that *zigA* is regulated by the transcriptional repressor Zur and environmental Zn^2+^ (4, 5, 24, 27, 32).

To further test the hypothesis that *S. aureus zigA* is responsive to Zn^2+^, bacteria grown in nutrient-rich tryptic soy broth (TSB) were either exposed to TPEN (*N*^1^,*N*^1^,*N*^2^,*N*^2^-tetrakis[(pyridin-2-yl)methyl]ethane-1,2-diamine), a small molecule chelator of transition metals with a high affinity for Zn^2+^, or vehicle for 1 h. Samples were then collected for qRT-PCR (Fig. 1D), and *zigA* transcript abundance was compared across wild-type, ∆*zigA*, ∆*zur*, and ∆*zigA + P_lgt_ zigA* strains (Table 1) (5, 27). In response to TPEN alone, *zigA* expression in wild-type strains is induced ~100-fold. ∆*zigA* samples, where *zigA* is removed from the genome by homologous recombination, exhibited transcript abundance consistent with the complete absence of *zigA* (Fig. 1D). Vehicle-treated samples of ∆*zigA + P_lgt_ zigA*, which were predicted to express *zigA* constitutively, experienced a ~20-fold average induction of *zigA*, whereas ∆*zur* samples demonstrate a >100-fold induction of *zigA*, consistent with *zigA* repression by Zur (Fig. 1D).

**TABLE 1:** Bacterial strains created/used

Bacterial strain or plasmid	Purpose, feature, and genotype	Source
*Escherichia coli* DH5α	Cloning strain	43
*Staphylococcus aureus* Newman	Wild-type, methicillin-sensitive clinical isolate	44
*S. aureus* RN4220	Restriction-deficient cloning intermediate strain	45
*S. aureus* RN9011	RN4220 harboring the pRN7023 integrase plasmid	46
pJC1306	Plasmid used for genomic integration into the *S. aureus* SaPI1 attachment site	46
Newman ∆*zigA*	In-frame unmarked deletion of *zigA* (*NWMN_0417; NWMN_RS02350*)	This Study
Newman ∆*zur*	In-frame unmarked deletion of *zur*	5
Newman *uvrA::erm*	*uvrA*::*erm* from the Nebraska Transposon Mutant Library transduced into Newman	This study; (47, 48)
Newman *katA::erm*	*katA::erm* from the Nebraska Transposon Mutant Library transduced into Newman	49, 50
Newman SaPI1::*P_lgt_-zigA-yopE*	Newman with constitutively active promoter controlling *zigA* expression integrated into the chromosome using the pJC1306 vector	This Study
Newman ∆*zigA* SaPI1::*P_lgt_-zigA-yopE*	Newman ∆*zigA* with constitutively active promoter controlling *zigA* expression integrated into the chromosome using the pJC1306 vector	This Study
Newman ∆*zigA* SaPI1::*P_lgt_-zigA-K17R-yopE*	Newman ∆*zigA* with constitutively active promoter controlling *zigA* with Lys to Arg point mutation at AA 17 expression, integrated into the chromosome using the pJC1306 vector	This Study
Newman ∆*zigA* SaPI1::*P_lgt_-zigA-C70A-yopE*	Newman ∆*zigA* with constitutively active promoter controlling *zigA* with a Cys to Ala point mutation at AA 70 expression, integrated into the chromosome using the pJC1306 vector	This Study
DH5α pBG102-*zigA*	Expression of recombinant ZigA with a C-terminal SUMO-His tag under Kanamycin resistance cassette	This Study
DH5α pBG102-*zigA-K17R*	Expression of recombinant ZigA with Lys to Arg point mutation at AA 17 and a C-terminal SUMO-His tag under Kanamycin resistance cassette	This Study
DH5α pBG102-*zigA-C70A*	Expression of recombinant ZigA with a Cys to Ala point mutation at AA 70 and a C-terminal SUMO-His tag under Kanamycin resistance cassette	This Study
DH5α pBG102-*uvrA*	Expression of recombinant UvrA with a C-terminal SUMO-His tag under Kanamycin resistance cassette	This Study
DH5α pBG102-*uvrA- Zn^2+^-binding domain*	Expression of recombinant UvrA AA 665-866 with a C-terminal SUMO-His tag under Kanamycin resistance cassette	This Study
DH5α pKOR1-∆*zigA*; neighboring regions (~1 kb)	Intermediate cloning strain for deletion of *zigA* by homologous recombination	This Study
RN4220 pKOR1-∆*zigA*; neighboring regions (~1 kb)	Intermediate cloning strain for deletion of *zigA* by homologous recombination	This Study
DH5α pJC1306-*P_lgt_-zigA-yopE*	Intermediate cloning strain for chromosomal integration of *zigA* under the *lgt* promoter	This Study
DH5α pJC1306-*P_lgt_-zigA-K17R-yopE*	Intermediate cloning strain for chromosomal integration of *zigA* with Lys to Arg point mutation at AA 17 under the *lgt* promoter	This Study
DH5α pJC1306-*P_lgt_-zigA-C70A-yopE*	Intermediate cloning strain for chromosomal integration of *zigA* with a Cys to Ala point mutation at AA 70 under the *lgt* promoter	This Study
RN9011 pJC1306-*P_lgt_-zigA-yopE*	Intermediate cloning strain for chromosomal integration of *zigA* under the *lgt* promoter	This Study
RN9011 pJC1306-*P_lgt_-zigA-K17R-yopE*	Intermediate cloning strain for chromosomal integration of *zigA* with Lys to Arg point mutation at AA 17 under the *lgt* promoter	This Study
RN9011 pJC1306-*P_lgt_-zigA-C70A-yopE*	Intermediate cloning strain for chromosomal integration of *zigA* with a Cys to Ala point mutation at AA 70 under the *lgt* promoter	This Study

These transcriptional observations resulted in the hypothesis that ZigA protein abundance would be significantly altered by TPEN treatment in these strains. After growth in nutrient-rich TSB ± TPEN, ∆*zur* samples exhibit increased ZigA protein abundance as compared to WT (Fig. 1E and F). As anticipated, ZigA is not detected in ∆*zigA* samples (Fig. 1G and H). Wild-type *S. aureus* treated with 10 µg/mL TPEN displays an increased abundance of ZigA, and ∆*zigA + P_lgt_-zigA-yopE* samples expressed high levels of ZigA (Fig. 1G). These observations are associated with even loading across wells, as indicated by Sypro Ruby staining (Fig. 1F and H). In those samples that express ZigA highly (∆*zigA + P_lgt_-zigA-yopE* and ∆*zur*), ZigA abundance decreases upon addition of TPEN, potentially consistent with Zn^2+^ limitation disrupting protein stability or accumulation under these conditions. Taken together, these experiments confirm that *zigA* is Zur and Zn^2+^-regulated, as reported previously (5, 27). These data further support the model that *zigA* is a COG0523 family member regulated by environmental Zn^2+^ and Zur and therefore likely part of the YeiR or YciC COG0523 subfamilies (Fig. 1) (4).

### *In vitro* binding experiments reveal candidate ZigA clients

**Fig 2:**
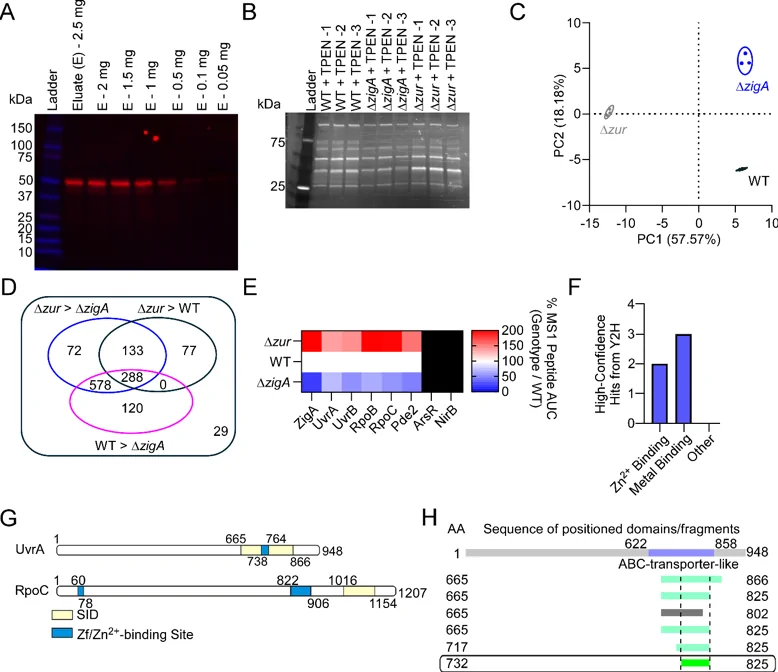
ZigA may interact with *S. aureus* UvrA in addition to candidate clients central to DNA damage repair and transcription. (**A**) Optimization of Co-IP conditions via application of a known quantity of beads to weight-matched samples of *S. aureus* lysate. Eluents from these parallel assays were visualized by immunoblot (primary Ab^o^: 1:10,000 Rabbit-poly-anti-*S. aureus* ZigA, secondary Ab^o^: 1:10,000 Goat anti-Rabbit Ig- IR 800CW; colorimetric blot imaging false colored to blue). (**B**) Sypro Ruby staining of triplicate Co-IP of WT, ∆*zigA*, or ∆*zur S. aureus* grown in TSB with 5 µg/mL of TPEN. (**C**) Principal component analysis (PCA) plot of MS1 data-independent analysis of Bruker timsTOF-MS describing the results of Co-IP of WT, ∆*zigA*, or ∆*zur S. aureus* grown in TSB with 5 µg/mL of TPEN. (**D**) Venn diagram depicting the number and association of hits observed from co-immunoprecipitation experiments. (**E**) Select protein complexes/operons significantly enriched in ZigA-expressing strains by co-IP also identified by Y2H or in studies of other bacterial COG0523 proteins. (**F**) High-confidence hits from yeast-2-hybrid with *S. aureus* ZigA as a probe against an *S. aureus* library. (**G**) Predicted interaction domains for two Zn^2+^-binding candidate clients, with Zn^2+^-binding (light blue) and predicted interacting domain (selected interaction domains [SID]; yellow). (**H**) 1-by-1 style yeast-2-hybrid analysis of UvrA fragment tested against a ZigA-bound probe, highlighting the minimal binding fragment required for ZigA-UvrA interaction.

To test the hypothesis that ZigA interacts with other *S. aureus* protein(s), consistent with the activity of other bacterial COG0523 proteins in the YciC sub-cluster, co-IP experiments were performed (26). After confirming binding and pulldown of ZigA (45 kDa) following immunoprecipitation (Fig. 2A), triplicate co-IP samples were prepared from wild-type, ∆*zigA*, and ∆*zur* cells treated with TPEN to enrich for ZigA. Total protein from pulldowns was detected using Sypro Ruby stain. After normalizing by total sample input weight, comparisons revealed that ∆*zigA* samples pulled down less protein than wild-type samples (Fig. 2B). Observation of lower pull-down yield when ZigA abundance is decreased supports the specificity of the developed α-ZigA antibody and the binding interaction.

Proteomic interrogation of co-IP samples by principal component analysis (PCA) of all three biological replicates from each of three strains (Fig. 2C; Fig. S2A) shows that over 70% of the variation between samples is explained by PC1 and PC2. PCA suggests that samples are predominantly separated by genetic background (Fig. 2C). Statistically significant differences in peptide MS1 integrated density are detailed by genetic background in Fig. 2D and Fig. S2. Those proteins enriched in WT and ∆*zur* samples compared to ∆*zigA* samples were annotated using the *S. aureus* Newman genome (866 protein IDs; UniProt Taxon ID: 426430). Prioritization of candidate hits depended on published findings surrounding COG0523 proteins, including eukaryotic ZNG1, *B. subtilis* ZagA, and *A. baumannii* MigC, as these proteins mediate post-translational changes in client activity (26, 28, 31, 36). We hypothesized that candidate interaction partners of ZigA may be enriched within protein complexes or members of an operon where each encoded protein interacts with ZigA. As such, the integrated DIA density relative to WT of co-IP hits and their operons (Fig. 2E) and/or protein complexes (Fig. S2C) are presented. *zigA* is regulated in response to Zn^2+^ abundance (Fig. 1D through H), suggesting a candidate client might require Zn^2+^ for function. Co-immunopreciptation revealed multiple candidate client proteins, including UvrA, RpoC, GyrB, LexA, SarA, ClpP, and PurR (Fig. S2), supporting a putative biological interaction between ZigA and any of these factors. Notably, UvrA and RpoC require zinc for function (51–54).

As an orthogonal approach to screen for ZigA interacting partners, Y2H experiments were performed using a construct expressing full-length *S. aureus* ZigA to identify candidate interacting partners (Fig. 2F and G; Fig. S3) (55). Screening against a *S. aureus* protein fragment library revealed that ZigA may have high-confidence interactions with *S. aureus* UvrA, RpoC, Pde2, ArsR, and NirB. Further Y2H domain mapping of the high-confidence candidate clients was performed to identify selected interaction domains (SID; yellow boxes in Fig. 2G). Focusing on predicted Zn^2+^-binding regions in the candidate clients (teal boxes in Fig. 2G), a high-confidence SID was predicted in a UvrA domain containing a Zn^2+^-binding site.

To determine whether the predicted SID is sufficient for interaction with ZigA, 1-by-1 Y2H experiments of *S. aureus* UvrA fragments surrounding this site were conducted (Fig. 2H) (55). These results suggest that the smallest fragment of UvrA that interacts with ZigA with high confidence is residues 732–825, which contains a predicted Zn^2+^-binding site comprised of four Cys residues (Fig. 2H). To prioritize further studies into candidate ZigA clients, Y2H hits were cross-referenced with those of co-immunoprecipitation experiments. ArsR and NirB were not differentially abundant within the co-immunoprecipitation experiments (Fig. 2E). Pde2, a candidate client with a role in the response to cell wall and oxidative stress, exhibits low abundance in ∆*zigA* samples and high abundance in ∆*zur* samples by co-IP, consistent with a potential ZigA-Pde2 interaction. We hypothesized Fol/Thy and Mur family proteins may be putative *S. aureus* ZigA interacting partners based on the roles of *B. subtilis* ZagA and *A. baumannii* MigC, which are implicated in the regulation of FolE and MurD, respectively (26, 36). MurD, FolE, and ThyA are in low abundance within ∆*zigA* samples and high abundance within ∆*zur* samples, mirroring the observed abundance of ZigA in these conditions and consistent with the identification of Mur, Fol, ThyA, and Pde2 proteins as candidate clients of *S. aureus* ZigA (Fig. 2; Fig. S2B and C) (56). Taken together, these data are consistent with a potential interaction between *S. aureus* ZigA and multiple stress response proteins.

### *S. aureus* lacking *zigA* exhibits small defects in growth and survival in low zinc, genotoxic environments

**Fig 3:**
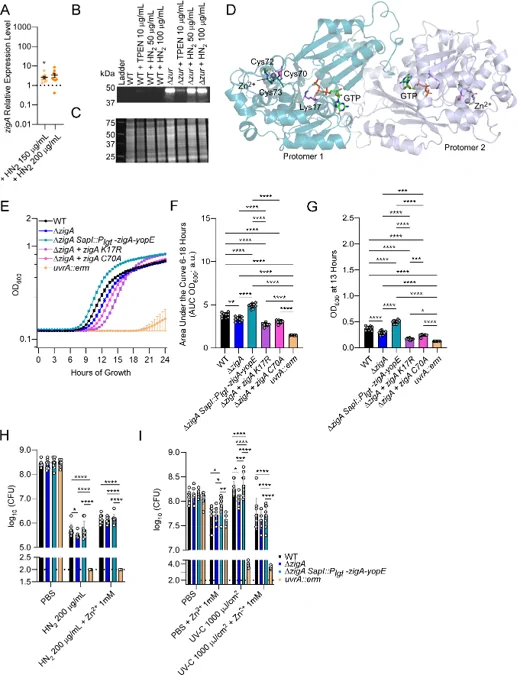
Loss of *zigA* is associated with *S. aureus* growth and survival defects in low Zn^2+^ and DNA-damaging environments. (**A**) Relative expression level (2^−∆∆Ct^) by SYBR Green-based qRT-PCR of *zigA* one hr after treatment of *S. aureus* with mechlorethamine. (**B**) After growth for 24 h in TSB with or without Zn^2+^ chelator TPEN, the abundance of ZigA (~45 kDa) was measured via Immunoblot using Rabbit polyclonal α-ZigA 1:5,000 in 1× TBST Blocking buffer with 5% (wt/vol) EasyBlocker. Secondary antibody staining was completed using Goat α-Rabbit Ig 1:10,000, then imaged on a Bio-Rad Chemi-Doc. (**C**) A parallel gel was used for Sypro Ruby staining as a measure of loading control (**B**). (**D**) Alphafold v.3.0 model of dimeric ZigA based on sequence homology with labels for the three predicted Cys residues in the CXCC Zn^2+^-binding site and K17 near the GTP-binding site. (**E**) Twenty-four-hour growth assays of *S. aureus* with and without *zigA* and residues predicted to be relevant to GTP binding (K17R) and Zn^2+^ binding (C70A), as well as a transposon mutant from the NTML library of *uvrA* treated with 150 µg/mL HN_2_ and 5 µg/mL TPEN. (**F**) Area under the curve (AUC) in the 12 h surrounding the exponential phase of growth seen in panel **E**. In turn, panel **G** describes the values of replicate curves at selected timepoints. Panels **E–G** are the result of triplicate biological replicates and nine total replicates, with statistics by ordinary one-way analysis of variance with Tukey’s multiple comparison test, with a single pooled variance. All presented distributions are mean ± standard deviation. (**H and I**) Survival of bacteria with and without *zigA* or *uvrA* was measured in the setting of genotoxins mechlorethamine (HN_2_, **H**) or UV-C, with and without supplemented Zn^2+^ (**I**) before washing, plating in a dilution series, and enumerating of surviving colonies. *P < 0.05, **P < 0.01, ***P < 0.005, and ****P < 0.001. Panels **B and C **do not include all comparisons to allow for effective visualization.

UvrA is critical to the intrinsic and extrinsic DNA damage response, including the response to UV damage and mechlorethamine (HN_2_) (57). To test the hypothesis that UvrA-associated stress may increase ZigA abundance, transcriptional and translational assays were conducted. HN_2_ treatment moderately induces *zigA* by qRT-PCR (Fig. 3A). However, this transcriptional phenotype does not translate to ZigA protein abundance by immunoblot (Fig. 3B and C). Strains overexpressing *zigA* exhibit a modest, but significant increase in *uvrA* transcription, even in the absence of HN_2_ (Fig. S4A). Transcription of *uvrA* is also significantly increased in TPEN or HN_2_-treated *S. aureus*, suggesting that co-IP experiments may be confounded by transcriptional changes associated with the tested conditions, which could artificially increase protein abundance in WT and ∆*zur* samples (Fig. 3A; Fig. S4A).

To extend this hypothesis and determine whether the loss of *zigA* phenocopies the loss of *uvrA*, growth assays were conducted. Measurements of growth in TSB alone, HN_2_, or TPEN stressed environments revealed that loss of *zigA* is not detrimental to growth in a nutrient-rich or single-stressor condition (Fig. S4B through J). Consistent with an impaired ability to resolve DNA damage, *uvrA* NARSA transposon mutants exhibited significant growth defects in each condition (Fig. S4) (47). ∆*zigA + P_lgt_ zigA* was associated with increased *uvrA* abundance (Fig. S4A) and a growth advantage in every tested condition (Fig. S4).

Strains harboring constitutively expressed *zigA* with mutations of the CICC Zn^2+^-binding site and within the Walker A motif were made (27, 33, 39, 41). The C70A mutation, which removes one of the Zn^2+^ chelating side chains, is expected to hinder transition metal binding in the high affinity CICC site and thereby reduce the structural stability of the protein, whereas the K17R mutation likely alters access to, or the structure of, the GTP-binding site and is expected to hinder nucleotide binding (Fig. 3D). These point mutations resulted in growth defects in HN_2_-treated conditions more than the loss of *zigA*, potentially resulting from these mutations interfering with as-yet-undefined protein-protein interaction(s) (Fig. S4H through J) (33, 41).

In combined HN_2_ and TPEN stress conditions (Fig. 3E through G), a modest but reproducible defect in growth is observed for ∆*zigA*. This defect is complemented by ∆*zigA + P_lgt_ zigA* and is recapitulated with C70A or K17R point mutations (Fig. 3E). There is a large growth defect associated with the loss of *uvrA* in *S. aureus*, consistent with the compromise of nucleotide excision repair known to affect the growth of *Escherichia coli* (Fig. S4) (51, 53, 57, 58). The *zigA*-dependent defect in growth requires the presence of 5 µg/mL TPEN and 150 µg/mL HN_2_ (Fig. 3E). Area under the curve (Fig. 3F) and OD_600_ measurement of replicates at mid-logarithmic growth phase of WT (Fig. 3G) support these observations.

The impact of ZigA on bacterial survival of UvrA-dependent DNA damage repair was further investigated through survival assays (Fig. 3H and I). Bacteria in the mid-log phase of growth exposed to PBS exhibit no *zigA*- or *uvrA*-dependent defects in survival. Treatment with HN_2_ elicited a statistically significant defect in the survival of strains lacking *zigA* or *uvrA* (Fig. 3H). Samples containing *uvrA* survive 100,000-fold more than those without. A statistically significant defect in survival associated with loss of *zigA* is rescued by ∆*zigA + P_lgt_-zigA* or with the addition of 1 mM ZnCl_2_ to the survival challenge (Fig. 3H). Similar findings were obtained when all strains were exposed to UV-C irradiation (254 nm; 10,000 µJ/cm^2^; Fig. 3I). UV-naïve samples exhibit no difference in survival associated with ∆*zigA* or *uvrA::erm*. Exposure to UV-C was associated with significant *zigA*-dependent and *uvrA*-dependent survival defects, rescued by *zigA* complementation or by Zn^2+^ supplementation (Fig. 3I). Therefore, ZigA plays a statistically significant role in *S. aureus* growth and survival in Zn^2+^-deplete and DNA-damaging conditions. However, the comparatively minimal impact of loss of *zigA* or *uvrA* on growth in the presence of DNA-damaging agents is inconsistent with a biologically relevant interaction between ZigA and UvrA.

### ZigA-dependent defects in *S. aureus* growth and survival appear to be independent of other transition metals and other known COG0523 protein client homologs

**Fig 4:**
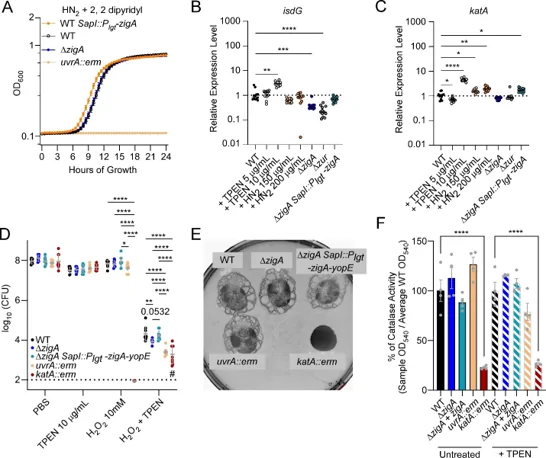
Effects of *zigA* are incompletely described by *katA,* other COG0523 interactors, or environmental iron availability. (**A**) Twenty-four-hour growth assays of *S. aureus* with and without *zigA* or *uvrA* in the presence of 150 μg/mL HN_2_ and 250 µM 2,2 dipyridyl. Relative expression level (2^−∆∆Ct^) by qRT-PCR of (**B**) *isdG* and (**C**) *katA* 1 h after treatment. Statistics for (**B and C**) using Kruskal-Wallis tests with post-hoc uncorrected Dunn’s test for each comparison to NWMN alone. (**D**) Survival assays of *S. aureus* with and without *zigA*, *uvrA*, or *katA*, in conditions of hydrogen peroxide and TPEN stress for 1 h. (**E**) Qualitative methods via bubbling secondary to H_2_O_2_ exposure when grown in TSA plates containing TPEN. (**F**) Evaluation of catalase activity in a quantitative (Catalase Assay Kit) manner from stationary phase cultures grown in nutrient-rich media with and without TPEN treatment. Statistics completed for panels **D and F** via ordinary two-way analysis of variance with Tukey’s multiple comparisons test with a single pooled variance. Biological triplicate data presented for all experiments. Indicated by #, all counted surviving colonies represent non-stable small colony variants. *P < 0.05, **P < 0.01, ***P < 0.005, and ****P < 0.001.

The modest overall impact of *zigA* on *S. aureus* growth and survival led to further investigation to determine whether other metals, interacting partners, or stresses may reveal more pronounced ZigA-dependent phenotypes. Growth in the setting of HN_2_ and 2,2,dipyridyl was used to test the hypothesis that iron is associated with *zigA*-dependent defects in growth. While *uvrA::erm* strains are unable to grow in this dually stressed condition, ∆*zigA* strains do not exhibit growth defects compared to WT, suggesting *zigA* phenotypes are independent of iron limitation (Fig. 4A). This is also reflected in the attenuation of ∆*zigA + P_lgt_-zigA* growth advantage in low iron compared to low zinc conditions (Fig. 3E and 4A). These data suggest that *S. aureus* growth in low iron is independent of *zigA*, consistent with ZigA exhibiting poor iron binding (27).

Other tested hypotheses included the potential for ZigA to have a functional interaction with Pde, Mur, Fol, or Thy family proteins, which would likely result in defects associated with cell wall stress or thymine starvation (26, 36, 37, 56). Application of TPEN and carbenicillin (Fig. S5A) or trimethoprim-sulfamethoxazole (known as bactrim or TMP-SMX; Fig. S5B) demonstrated no growth defects associated with ∆*zigA*. This lack of defect was further demonstrated by antimicrobial susceptibility E-testing, where there are no significant changes in TMP-SMX susceptibility associated with the loss of *zigA* with or without TPEN co-treatment (Fig. S5C and D). Multiple CLSI-approved antimicrobial susceptibility testing methods show that while *uvrA::erm* maintains expected erythromycin and clindamycin resistance, there were no other significant changes in antimicrobial susceptibility among strains lacking *zigA* or *uvrA* (Fig. S5C). The persistent lack of differences in *S. aureus* sensitivity to these antibiotics suggests that any interaction between ZigA and ThyA, Pde2, Fol, or Mur family proteins is not biologically significant.

**Fig 5:**
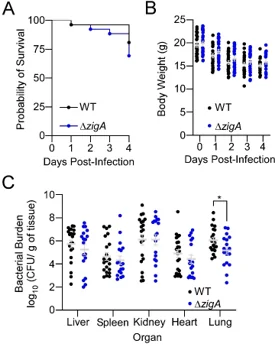
ZigA confers an advantage to *S. aureus* during systemic infection, specifically within the lung. (**A**) From mice infected with 2 × 10^7^ *S. aureus*, mortality over the 4-day infection period as evaluated by log-rank Mantel-Cox test. (**B**) Changes in weight (g) of infected C57BL6/J mice as evaluated by two-way analysis of variance (ANOVA) with Fisher’s uncorrected LSD. (**C**) log_10_ transformed measures of CFU per gram of tissue after 4 days of infection with either Newman or *ΔzigA*, analyzed by two-way ANOVA with Fisher’s uncorrected LSD. All comparisons are not significant unless otherwise noted. *P < 0.05.

Candidate client RpoC, and the activity thereof, is associated with resistance to antimicrobials, including rifampicin (59). *E*-tests for rifampicin were conducted on plates with and without TPEN, and susceptibilities were not significantly affected by the loss of *zigA* (Fig. S5D). These experiments reduce confidence that a predicted interaction between RpoC and ZigA in *S. aureus* results in phenotypes consistent with a *rpoC* mutant (59). Collectively, these data do not reveal significant *zigA*-dependent phenotypes.

It is also possible that the similar transcriptional profile identified between *zigA* and *uvrA* confounds the results of co-IP. Hence, candidate ZigA clients identified by co-IP but not yeast-2-hybrid were prioritized, including those with *S. aureus* known/expected roles in transition metal homeostasis and/or DNA damage stress (Fur, Isd, and Lex family proteins). It was predicted that the transcription of these candidate clients would correlate with transcription of *zigA*, similar to *uvrA* (Fig. 1D; Fig. S4A). To measure the effect of *zigA* on transcription of additional candidate clients, qRT-PCR was completed for *fur* (Fig. S4E), *isdG* (Fig. 4B), and *lexA* (Fig. S4F). While *fur* expression was induced by TPEN and HN_2_, there was no change in *fur* abundance in ∆*zigA* or ∆*zigA + P_lgt_ zigA* (Fig. S4E). *isdG* transcription is Fur-dependent and was induced by TPEN, but not HN_2_. ∆*zur* and ∆*zigA* strains downregulate *isdG*. This phenotype was recovered in ∆*zigA + P_lgt_ zigA* (Fig. 4B) (60). In the absence of iron-dependent growth phenotypes, this correlation between *isdG* and *zigA* transcription may instead reflect conserved systems wherein *S. aureus* adapts to multi-metal chelation at the host-microbe interface (5, 61–63). As expected, SOS transcription factor *lexA* was induced only in the setting of HN_2_ treatment (Fig. S4F) (64). These data do not support the interaction between ZigA and Fur, LexA, IsdG, or iron in the tested conditions.

### ZigA appears to promote survival in Zn^2+^-deplete conditions within multiple genotoxic environments

Co-immunoprecipitation also identified catalase (KatA; Fig. S2C) as a priority candidate client that may interact with ZigA. Catalase is a heme-dependent virulence factor that confers H_2_O_2_ and DNA damage resistance (65, 66). To test the hypothesis that ZigA may interact with KatA, transcriptional and survival studies were conducted. In contrast to transcription of *lexA*, *katA* transcript abundance increases significantly in the setting of Zn^2+^ limitation, HN_2_ exposure, or in ∆*zigA P_lgt_-zigA* samples (Fig. 4C). This coincident transcriptional upregulation was reminiscent of that observed between *zigA* and *uvrA* (Fig. 1D; Fig. S4A). To determine whether KatA and ZigA may interact, survival assays were completed. When exposed to hydrogen peroxide alone, *katA* transposon mutants exhibit a million-fold decrease in survival compared to WT strains (Fig. 4D). WT cells experience a thousand-fold decrease in survival in the setting of TPEN and H_2_O_2_ treatment as opposed to H_2_O_2_ alone. Strains without *zigA*, *katA*, or *uvrA* suffer a significant survival defect in this dually treated condition as compared to WT (Fig. 4D). This modest *zigA*-dependent defect coincides with a much larger *uvrA*-dependent survival defect, which is rescued in ∆*zigA + P_lgt_ zigA* strains that have increased *uvrA* and *katA* abundance (Fig. 4A through D). Qualitative (Fig. 4E) and quantitative (Fig. 4F) catalase activity measurements show no association between KatA activity and the presence of *zigA*, suggesting the putative KatA-ZigA interaction is not biologically relevant.

### ZigA does not determine the outcomes of murine retro-orbital systemic *S. aureus* infection

To test the hypothesis that ZigA participates in staphylococcal pathogenesis *in vivo*, 6-week-old C57BL6/J mice were subjected to retro-orbital systemic infection with wild-type or ∆*zigA S. aureus*. After 4 days, major organs were collected and homogenized for CFU enumeration. Infection with *∆zigA* was not associated with significant changes in mouse mortality or weight loss (Fig. 5A and B). The only observable phenotype from the measures collected was a modest defect in pulmonary bacterial burden (Fig. 5C). Overall, it appears that any role of *zigA* in *S. aureus* infection in this murine model is limited.

## DISCUSSION

COG0523 proteins have garnered significant interest as they participate in metal homeostasis across the tree of life (28, 30, 31). Nutrient trace transition metals form a basis for competition at the host-microbe interface through a process termed nutritional immunity (6, 10, 13–15). In staphylococcal biology, transition metals, including Zn^2+^, are critical to bacterial growth and pathogenesis (5, 11, 67–71). *S. aureus* has three COG0523 proteins, including ZigA, which led to this investigation surrounding the biochemistry, microbiology, and pathogenic impact of ZigA in this priority pathogen.

Size-exclusion chromatography revealed that the presence of GTP causes ZigA to shift to a homodimeric state (Fig. 1A; Fig. S1). Evaluation of GTPase activity confirmed that ZigA has intrinsic GTPase activity and showed that this activity is stimulated by Zn^2+^ (27). In the presence of Zn^2+^, the GTP to GDP *V*_max_ increased by almost 40% and *K*_*m*_ decreased by over 45% (Fig. 1B). This acceleration in GTPase activity is reversed by the Zn^2+^ chelator EDTA, consistent with the previous proposal that ZigA GTPase activity is Zn^2+^ dependent (26, 32, 33, 72). Directly upstream of *zigA* is a putative Zur box (Fig. 1C) (4, 5, 24). Studies of *zigA* transcription (Fig. 1D) and protein abundance (Fig. 1E through H) showed that, consistent with the literature, *zigA* is regulated by Zur, and its abundance is inversely correlated with environmental Zn^2+^ (5, 27). This conclusion matches the phylogenetic categorization of ZigA as a functional dimer and part of the Zn^2+^-regulated YeiR/YciC sub-cluster of COG0523 proteins (4, 24, 26, 32, 33).

COG0523 proteins post-translationally regulate clients by coupling GTPase activity and protein interaction (26, 32, 73). Eukaryotic ZNG1, for instance, acts as a Zn^2+^ metallochaperone for METAP1, enabling METAP1 function across organisms (28, 30, 31). *A. baumannii* MigC interacts with MurD, compromising its activity and *A. baumannii* resistance to cell wall stress (36). *B. subtilis* ZagA interacts with FolE and acts as a Zn^2+^ metallochaperone in the setting of Zn^2+^ limitation (26). In the case of *S. aureus* ZigA, candidate metal-binding clients were identified by co-immunoprecipitation (Fig. 2A through E). Samples of *S. aureus* under *zigA*-inducing conditions exhibit an enrichment in UvrA, RpoC, KatA, and other DNA damage repair machinery in WT and ∆*zur* backgrounds as compared to ∆*zigA* backgrounds (Fig. 2E). These included members of the *uvr* and *rpo* operons, as well as proteins implicated in COG0523- or metal-associated phenotypes in other organisms, including FolE and MurD (Fig. 2D and E; Fig. S2) (5, 26, 65, 66). These hits were further prioritized via yeast-2-hybrid (Fig. 2F through H). The observed interaction between ZigA and UvrA (Fig. S3) was further probed in yeast 2-hybrid 1-by-1 assays (Fig. 2H). UvrA participates in the response to DNA damage repair, specifically in the initial steps of nucleotide excision repair, which is critical to overcome intra- and inter-strand DNA crosslinks (58). In *Escherichia coli*, UvrA binds DNA in a Zn^2+^- and ATP-dependent manner and recruits UvrB to sites of DNA damage, forming the basis of the Uvr endonuclease (51–53, 58). To test whether ZigA and UvrA may interact in *S. aureus*, we took a cell-based approach based on the prediction that in Zn^2+^-deplete environments, *S. aureus* lacking ZigA may phenocopy those lacking the native client of ZigA.

*S. aureus* at the host-microbe interface simultaneously experiences multiple host-derived stresses. Among these, metal limitation and DNA damage are common (7, 20, 22, 67, 74, 75). Mechlorethamine (HN_2_) chemically induces intra- and inter-strand DNA crosslinks, as does UV-C irradiation (51–53, 57). HN_2_ treatment resulted in increases in *zigA* transcription, but not ZigA translation (Fig. 3A and B). Growth curves of *S. aureus* with and without *zigA* suggest that the combination of Zn^2+^ chelation by TPEN and DNA damage by HN_2_ is sufficient to induce a small *zigA*-dependent defect in growth (Fig. 3E through G).

Based on the hypothesis that growth phenotypes may be an inadequate measure of the response to DNA damage by irreversible chemical crosslinking dosed at a single time point, this work turned to assays evaluating survival. Survival of *S. aureus* under HN_2_ or UV-C treated conditions results in a survival defect of less than one log_10_-fold dependent on *zigA*, which is ameliorated with the addition of exogenous Zn^2+^ to the survival challenge (Fig. 3H and I). These differences implicate ZigA in the response to DNA damage and low environmental Zn^2+^ (Fig. 3; Fig. S4). However, these *zigA*-dependent differences did not approach the magnitude of those associated with the loss of UvrA, including in combined Zn^2+^-deplete and genotoxic environments. In the absence of direct protein-protein biochemical studies probing the potential interaction between ZigA and UvrA, there is currently insufficient evidence to conclude that UvrA and ZigA interact in *S. aureus*. However, UvrA was only one of a set of possible interacting partners for ZigA (Fig. 2; Fig. S2 and S3).

RpoC, identified in Fig. 2E through G, was originally de-prioritized because the predicted ZigA-interacting domain by yeast-2-hybrid did not contain a metal-binding site. RpoC is critical to the response to rifampicin and is readily implicated in the development of rifampicin resistance (59). In the presence or absence of Zn^2+^ chelator TPEN, there are no *zigA*-dependent changes in susceptibility to rifampicin (Fig. S5). Indeed, there were no significant changes in antimicrobial resistance associated with ∆*zigA* or *uvrA:erm* except those associated with transposon mutagenesis (Fig. S5) (48). *S. aureus* strains were also exposed to TPEN and carbenicillin or trimethoprim-sulfamethoxazole to evaluate potential ZigA-dependent phenotypes tied to Mur or Fol proteins (26, 36, 37, 56). These stressors were not associated with a significant difference in growth or survival (Fig. S5A and B). *zigA*-dependent defects were not elicited using iron-specific chelator 2, 2, dipyridyl, consistent with previous work concluding ZigA does not bind iron (Fig. 4A) (27). Changes in *zigA* expression are poorly associated with changes in expression of iron homeostasis genes (Fig. 4B; Fig. S5E and F).

Catalase binds iron in the form of heme and was identified as a candidate client via co-IP (Fig. S2). However, *zigA* has no observable or quantifiable impact on KatA activity (Fig. 4E and F). ∆*zigA* exhibits a survival defect when exposed to TPEN and 10 mM H_2_O_2_, as do *uvrA::erm* strains and *katA::erm* strains (Fig. 4D). In that setting, the loss of *katA* results in ~2 log_10_-fold decrease in survival, whereas the loss of *uvrA* results in ~1 log_10_-fold decrease, and the loss of *zigA* results in ~0.5 log_10_-fold decrease.

While ZigA appears to participate in the response to low Zn^2+^ and multiple types of DNA damage, there were no discovered conditions where ZigA exhibits large growth or survival defects, which would be expected if ZigA interacts with and affects the activity of specific client proteins. Screening of hundreds of potential stressors derived from a screen of Biolog bacterial stressors (PM 11–20) in a Zn^2+^-deplete defined media yielded no reproducible growth defects in ∆*zigA* larger than that of H_2_O_2_ or HN_2_ treatment (data not shown). Since COG0523 proteins are predicted to be critical to the host-microbe interface and involved in pathogen transition metal utilization, systemic infection of mice with staphylococci ± *zigA* was completed, which suggests the loss of *zigA* does not impact animal survival or weight loss. However, the loss of *zigA* does mediate a modest defect in the pathogenesis of the lung (Fig. 5A and B). These observations may be attributable to differences in transition metal abundance or genotoxic stress in the lungs compared to other organs (31, 76, 77). It is also possible that despite investigating several candidate clients, there are as-yet-unidentified clients of ZigA that confer a survival/growth advantage to *S. aureus* in the pathogenesis of the lung.

Staphylococcal COG0523 proteins are currently understudied, despite the detrimental impact of *S. aureus* on human health. Expanding the available microbial genetic tools for the study of ZigA and putative interacting partners confirms the work of Jordan and colleagues, demonstrating that *S. aureus* ZigA is a *bona-fide* member of the COG0523 proteins regulated by Zur (5, 27). ZigA forms dimers in the presence of GTP, and intrinsic ZigA GTPase activity is accelerated in the presence of Zn^2+^. Taken together, in low Zn^2+^ environments, staphylococci increase the expression of ZigA, which dimerizes in the presence of GTP and likely interacts with Zn^2+^ and some unidentified protein client with broad roles in DNA damage repair. While the size of ZigA-dependent growth and survival phenotypes is modest, future work could consider the possibility of moonlighting or cross coverage between the three *S*. *aureus* COG0523 proteins. Evaluation of *S. aureus* lacking multiple COG0523 proteins may result in significant phenotypes and be used to probe the full impact of COG0523 proteins on *S. aureus* pathogenesis.

## MATERIALS AND METHODS

### Bacterial maintenance and construct creation

Bacterial strains (Table 1) and sequences for primers used (Table 2) define the name, features, and source of the obtained biological molecules. Following standard microbiological technique, bacterial inocula were prepared from the *S. aureus* strain Newman, and mutants were plated on tryptic soy agar plates and grown overnight. *Escherichia coli* cultures were grown in lysogeny broth (LB) or on LB agar. Transposon mutants derived from the Nebraska Transposon Mutant Library (NTML) were maintained on tryptic soy agar plates containing 5 µg/mL of erythromycin (47, 48). Single colonies from these plates were then inoculated into 5 mL of tryptic soy broth and grown at a 45° angle, 180 rpm, 37°C overnight in an Innova44 incubator (14 < *x* < 20 h).

**TABLE 2:** Primers used

Primer	Sequence	Source
Upstream	CTCGATTCTATTAACAAGGG	47
Buster	GCTTTTTCTAAATGTTTTTTAAGTAAATCAAGTAC	47
pJC1306-seqF	GCTCACATGTTCTTTCC	46
pJC1306-seqR	ATTATACATGTCAACGATAATAC	46
pJC1306-IntegrateF	GGTATTAGTTTGAGCTGTCTTGGTTCATTGATTGC	46
pJC1306-Integrate R	GTGCTTCACCAGCACCACATGCTG	46
pJC1306-Plgt-yopE-F	CAGGTCGACTCTAGAGGATCCACTAATGATTTATTATGTAGTGGTTCTTTG	This Study
pJC1306-Plgt-yopE-R	GAATAGGCGCGCCTGAATTCGCATGCTAAAAAACGATTCG	This Study
Plgt-zigA-F	AAATACAATTGAGGTGAACATATGATGGCTAAAATTCCAGTTACG	This Study
zigA-yopE-R	GCAAATTAGACCAGCACGAGGTGGTGGTG	This Study
ZigA-K17R-F	AGGCTCGGGGCGCACAACGTTGT	This Study
ZigA-K17R-R	AAATAACCACTTAATACCGTAAC	This Study
ZigA-C70A-F	TTCTAATGGTGCGATCTGTTGTACACTTAGAG	This Study
ZigA-C70A-R	AGTTCGACTAATTTTTCATCTG	This Study
pKOR-Seq-F	ATTGTCAGATAGGCCTAATGAC	This Study
pKOR-Seq-R	GCAATTAATGTGAGTTAGCTCA	This Study
ZigA-up-F	GACTCACTATAGGGGATATCTTATGAAAAGCAGTTAGATAAGC	This Study
ZigA-up-R	CATTCAACTTACATATATATACCCTCACTTCAATTTATTTG	This Study
ZigA-down-F	TATATATATGTAAGTTGAATGAAGTATAATCATTTTTG	This Study
ZigA-down-R	CCAGTCTTAAGCTCGGGCCCTAATCGAACAATTCACTGG	This Study
pBG102-Seq-F	TACGACTCACTATAGGGGAATT	This Study
pBG102-Seq-R	AGTTTGGAACAAGAGTCCACTA	This Study
pBG102-UvrA truncated-F	AGTTCTGTTCCAGGGGCCCGGGTAGAACGCCACGCTCTAATC	This Study
pBG102-UvrA truncated-R	CGACGGAGCTCGAATTCGGGTCCAAGACCAACATCAACTAGTG	This Study
UvrA616-846-F	ATGGGATCC GGAGCTAGAAGCAACAATCTTAAAG	This Study
UvrA616-846-R	ATGGAATTC TTATGAACGTTTATGAAGTTCAGATGC	This Study
rrsA-qPCR-F	GCACATCTTGACGGTACCTAAT	This Study
rrsA-qPCR-R	GCGCTTTACGCCCAATAATTC	This Study
znuC-qPCR-F	GTTGGACCAAATGGTGCTGG	This Study
znuC-qPCR-R	ACCTTCAACAAAAATCTCACCACT	This Study
uvrA-qPCR-F	ATCTGGGTCAGGTAAATCGTCATTAG	This Study
uvrA-qPCR-R	TTGACGCGCATAGGCACTTA	This Study
zur-qPCR-F	TCCTGGAATTTCATTCGACACAATA	This Study
zur-qPCR-R	CAAGCGATTCTAAACTTCATTTCACC	This Study
fur-qPCR-F	ACAACGCGAAGCTACTGTTAGA	This Study
fur-qPCR-R	AGCCAATTTCAGGCGCTTTATC	This Study
lexA-qPCR-F	TGGTGAAGCAGTTGGCTTAG	This Study
lexA-qPCR-R	ACGTGGTTTCGTTGGATCTC	This Study
zigA-qPCR-F	CAGCGATTTGCCGTTTAGATAC	This Study
zigA-qPCR-R	GCTTTGATCACGATCCATCAATAA	This Study

Cloning and mutagenesis experiments were confirmed using Sanger sequencing via GeneWiz and/or via whole-genome sequencing via Plasmidasaurus or SeqCenter as appropriate. To determine the effects of *zigA* on *S. aureus*, wild-type *S. aureus* Newman strain was subjected to homologous recombination to remove *zigA* from the chromosome (∆*zigA*; Table 1). To create strains for complementation, *zigA* was re-integrated into the genome of ∆*zigA* via pJC1306 under a constitutive *lgt* promoter and with a *yopE* terminator sequence (∆*zigA + P_lgt_ zigA*; Table 1) (46, 78). The corresponding mutations in integration constructs were made and integrated into the chromosome of *S. aureus* strains lacking *zigA* (∆*zigA* Sap1::*P_lgt_-zigA-K17R* and ∆*zigA* Sap1::*P_lgt_-zigA-C70A*; Table 1) (46).

Phusion 2× Hi-fidelity master mix was used for all non-quantitative PCRs. Plasmids for protein expression use a pBG102 backbone, which was linearized via BamHI and SalI, then assembled with fragments from *S. aureus* genomic DNA via NEBuilder HiFi DNA Assembly Master Mix. NTML transposons were transduced as described previously from a USA300 background to a Newman background and mapped using published primers Upstream and Buster as appropriate (47, 48). Chromosomal integration was conducted as described in Chen et al., using pJC1306 which incorporates into the *SapI* site of the Newman genome (46). Colonies were selected for using chloramphenicol and anhydrous tetracycline resistance. pJC1306 was linearized using SalI and BamHI, within which *P_lgt_-yopE* was inserted via HiFi DNA assembly. This base construct was linearized via NdeI, and constructs were completed via HiFi DNA assembly. Subsequent point mutagenesis, in cloning strains, was completed using the NEB Q5 SDM Kit according to the manufacturer’s instructions.

### Detailing of genomic context

Genomic context was derived from Aureowiki for *S. aureus* strain Newman then manually scaled (42). Upstream regions were collected from consensus Newman genome sequences, and then manually checked against the sequences of wild-type bacterial strains used in these studies (42). Predicted/known function of genes, as labeled, was inferred from the listed categories as deposited in Aureowiki (42). RBS and promoter regions, as well as the Zur box sequence, were determined from consensus sequences (79–81).

### Structure generation by AlphaFold 3.0

The structure of *S. aureus* ZigA homodimer was predicted by submitting the single-letter amino acid sequence to the AlphaFold3.0 server (82). Two copies of ZigA were specified along with one GTP and one Zn^2+^ ion per subunit. All jobs were run using default settings. The top-scoring model was selected for all analyses of these predicted structures. Confidence metrics for the top-scoring models are: ZigA homodimer (Fig. 3D) pLDDT = 78, pTM = 0.46, and pTM = 0.42.

### Immunoblotting

ZigA was heterologously expressed and purified from *E. coli* and used as an antigen to derive from rabbit polyclonal α-ZigA antibodies. Aerobic overnight cultures of strains of interest were sub-cultured 1:100 (vol/vol) in fresh media with or without stress, as listed per lane and per figure. Unless otherwise noted, all culture took place in a TSB background. Application of TPEN or HN_2_ stress was applied at the listed concentrations, as well as an equal volume of vehicle treatment to unstressed cultures. After sub-cultured cultures had grown for 24 h, cultures were centrifuged, and the media were discarded. Cell pellets were resuspended in 400 µL sterile PBS containing 10 mM MgCl_2_, then subjected to treatment with lysostaphin for 1 h at 37°C, 180 rpm. After treatment, samples were treated with final concentrations of 1% IGEPAL in PBS and 250 µM phenylmethylsulfonyl fluoride (PMSF). Once incubated on ice for 10 min, samples were transferred to 2 mL Lysing Matrix B tubes and subjected to three rounds of bead beating at a speed of 5.0 for 45 s, with incubation on ice between cycles (83). Samples were then treated with 20 µg of DNAse I, cell debris was pelleted, and supernatant containing protein was normalized for protein concentration via BCA and Sypro Ruby Staining.

At least triplicate biological samples normalized by protein concentration were then run on two parallel gels for 35 min at 200 V. The first was used for Sypro Ruby staining for total protein content, following the extended protocol provided by Thermo-Fisher (Catalog ID: S12000). The other gel was transferred to a nitrocellulose membrane for 18 min at 25 V and 1.0 A in a semi-wet BioRad system, then blocked using TBST w/ 5% (wt/vol) EasyBlocker (GeneTex Catalog No: GTX425858) for 120 < *x* < 180 min rocking at room temperature (84). The primary antibody was developed in collaboration with Thermo-Fisher (Project #1YI1840S). The developed antibody has a >1:204,800 affinity as measured by enzyme-linked immunosorbent assay for *S. aureus* ZigA per Thermo-Fisher report (data not shown). This antibody was then applied at the listed concentration per figure at 4°C rocking overnight (16–18 h), then washed with TBST alone in three 5 min cycles, and treated with secondary antibody as identified per figure for 2–3 h rocking at room temperature. Blots were then imaged in colorimetric and fluorescent channels relevant to the secondary antibody of interest on a BioRad ChemiDoc imaging system. All displayed immunoblots are representative of the collected data from >3 samples across three or more immunoblots/stained gels.

### Expression and purification of ZigA

His-tagged *S. aureus* ZigA was over-expressed at 37°C in *Escherichia coli* C41 (DE3) cells cultured in LB medium containing 100 µg/mL kanamycin. When the culture reached an OD600 of ~0.6, protein production was induced with 1 mM isopropyl-β-d-thiogalactopyranoside and continued for 4 h at 37°C before the cells were harvested by centrifugation (7,000 × *g*, 30 min, 4°C). Pelleted cells were resuspended in ice-cold lysis buffer (20 mM Tris-HCl, pH 8.0, 150 mM NaCl, 40 mM imidazole, 1 mM PMSF, and 1 mg/mL lysozyme) and lysed by sonication on ice (10 min total on-time, 10 s on/10 s off, 60% amplitude). The lysate was clarified by ultracentrifugation (20,000 rpm, 45 min, 4°C, Ti-45 rotor) and the supernatant loaded onto a 5 mL HisTrap FF column (Cytiva) equilibrated with lysis buffer. After washing with 20 column volumes of the same buffer, the bound protein was eluted with a linear 0–500 mM imidazole gradient. Elution fractions containing ZigA were pooled, supplemented with SUMO‐specific His-tag protease (3 U/mg protein), and dialyzed overnight at 4°C against the lysis buffer to remove excess imidazole and permit tag cleavage. The digest was reapplied to a second HisTrap column; the cleaved, tag-free ZigA appeared in the flow-through while the His-tag and protease remained bound. DTT (1 mM) and EDTA (2.5 mM) were added to the collected flow-through, and the solution was concentrated to ~1 mL with a 15 kDa MWCO centrifugal filter (Amicon Ultra). The protein was further purified by size-exclusion chromatography on a Superdex 200 10/300 GL column (20 mM Tris-HCl, pH 8.0, 150 mM NaCl, 2 mM EDTA, 1 mM DTT; 0.3 mL/min). Fractions corresponding to monomeric ZigA were pooled, concentrated to 5–10 mg/mL, flash-frozen in liquid nitrogen, and stored at −80°C. The final preparation routinely afforded >95% pure ZigA, suitable for biochemical studies. To study the effect of GTP on the oligomeric state of ZigA, GTP was added to a final concentration of 200 µM to the monomer ZigA peak (2 mg/mL), followed by incubation on ice for 2 h before loading onto the pre-equilibrated Superdex 200 Increase 10/300 GL column (20 mM Tris-HCl, pH 8.0, 150 mM NaCl, 2 mM EDTA, and 1 mM DTT).

### Evaluation of GTPase activity

Similar to that reported in Weiss and Murdoch et al., malachite green assays were performed using kit MAK307 from Sigma Aldrich (31). Reactions were run in 150 mM NaCl, 20 mM Tris-HCl, 1 mM DTT, and 2 mM MgCl_2_. A total of 100 µL reactions of 2 µM ZigA in the presence or absence of 2 µM Zn^2+^ or 2 µM Zn^2+^ plus 0.5 mM EDTA were initiated with the addition of GTP (0, 30, 50, 80, 100, 150, 200, 250, 300, 400, 500, and 600 µM) and incubated at 37°C, shaking, for 120 min. GTPase reactions were quenched with 150 µL of milliQ water, and inorganic phosphate produced was detected with the addition of 20 µL of working reagents from the kit (100 volumes of reagent A and 1 volume of B) (85). This detection reaction was incubated at room temperature for 30 min. Inorganic phosphate concentration was calculated based on the absorbance at 620 nm relative to a standard curve. Three biological replicates were acquired for each condition. The rate of reaction and Michaelis-Menten kinetics were calculated by fitting the data to standard equations in GraphPad Prism (version 10.4.2), and values are reported as mean ± 95% CI (86).

### Yeast-2-hybrid assays

Yeast-2-hybrid assays were performed by Hybrigenics Services, where full-length ZigA was used as bait against a library of staphylococcal protein fragments. Quality and number of potential interacting partners identified as hits were defined by Hybrigenics protocols (28, 31). In follow-up, a 1-by-1 assay was conducted where the interacting region of *S. aureus* UvrA (AA 665–866) was progressively made smaller to determine the minimal required interaction domain with *S. aureus* ZigA (55, 87).

### Co-immunoprecipitation of ZigA

This approach made use of the Dynabead co-immunoprecipitation kit and Dynabeads M-270 Epoxy beads (Thermo-Fisher; Cat. 14321D) with an antibody developed in rabbit specific to *S. aureus* ZigA (Thermo Fisher; Project #1YI1840S). Antibody was loaded onto beads at a ratio of 10 µg/1 mg of beads overnight following manufacturer instructions (https://assets.thermofisher.com/TFS-Assets/LSG/manuals/dynabeads_coimmuno_man.pdf). Aerobic overnight cultures of strains of interest were sub-cultured 1:100 (vol/vol) in 100 mL of fresh TSB with 10 µg/mL of TPEN in triplicate, and after 24 h of culture, cultures were spun down at 4,000 × *g* and subjected to cryolysis per Thermo-Fisher protocol. Freshly prepared extraction buffer containing 20 mM HEPES (pH 7.5), 150 mM NaCl, 2 mM TCEP, one crushed cOmplete mini protease inhibitor tablet, and kit IP buffer was used to extract proteins and prepare for co-immunoprecipitation. Each sample comprised at least 1 g of bacterial pellet, which was then subjected to extraction, wash, and elution in freshly prepared buffers as prescribed by kit manufacturers, including the use of volatile HPH elution buffer. Samples were then sent to the Vanderbilt Mass Spectrometry Research Center, where they were prepared for independent analysis by trypsin digesting the protein mixtures using S-traps (Protofi) according to the manufacturer’s recommended protocol. Resulting peptides were separated via a 30 min aqueous to organic gradient on a PepSep column (25 cm by 75 micron, 1.5 micron particle size) using a NanoElute II microflow HPLC system, and data were acquired on a Bruker timsTOF-Ultra using DIA-PASEF (Data-Independent Acquisition with Parallel Accumulation–Serial Fragmentation). Analysis of resulting data was performed across triplicate measurements per genotype using Biognosys Spectronaut v.19.5.241126, using MS1 quantification and tested for differential abundance by unpaired *t*-test.

### Growth curves

Overnight cultures were sub-cultured 1:100 in 5 mL of tryptic soy broth and cultured under the same conditions as stated previously for 60–90 min, then cells were pelleted at 4,000 × *g* for 10 min unless otherwise noted. Supernatant was disposed of, and the bacteria were then normalized to an OD_600_ of 1.0 in sterile PBS before being diluted 1:500 into culture media. When appropriate, TPEN (N,N,N′,N′-Tetrakis[2-pyridylmethyl]ethylenediamine) obtained from Millipore Sigma (Cat. No. AAJ64206EXH) was used at concentrations of 5 or 10 µg/mL, and HN_2_ (Mechlorethamine) obtained from Millipore Sigma (Cat. No. 122564) was used at 150 and 200 µg/mL as indicated per figure captions. One hundred microliters of culture media with cells were inoculated into 96-well, clear, flat-bottom plates from CytoOne, and diluted 1:1 with culture media containing the desired concentration of stressor. OD_600_ measurements were then made every half hr for ~24 h using a protocol involving continuous orbital shaking at 37°C on an EPOCH spectrophotometer.

### Survival assays

Tests of bacterial survival were conducted using a stress-and-recover model, where inocula are prepared, exposed to a set of stressors, incubated for short periods of time (30–60 min), and plated on rich media for recovery and quantification (88). Overnight cultures were sub-cultured 1:100 and grown for 3 h at a 45° angle, 180 rpm, 37°C. After that time, pellets equivalent to OD_600_ = 0.5 in 1 mL of sterile PBS were resuspended in 1 mL sterile PBS with and without stressors. These exposed cultures were then incubated for 30 min (HN_2_-exposed cultures and controls) or 60 min (all other conditions). ZnCl_2_ was obtained from Sigma (Cat. No. 208086), H_2_O_2_ from VWR (Cat. No. 95321), and PBS from ThermoFisher (Cat. No. 14190144). After incubation, cells were serially diluted in 96-well, clear, flat-bottom CytoOne plates from 10^−2^ to 10^−8^ dilution. Droplets of the dilution series were dropped onto nutrient-rich TSA plates, thrice per biological replicate per condition. Droplets were allowed to dry under flame in closed petri dishes obtained from Fisher (Cat. No. FB0875712). In UV-C exposed survival assays, plates were placed open face into a Stratagene UV Stratalinker 2400 and exposed to 10,000 µJ/cm^2^ of UV-C radiation at 254 nm (89, 90). Plated dilution series were grown overnight (14 < *x* < 24 h), and colonies were enumerated at the highest level of dilution where colonies were countable, prioritizing dilutions where between 3 and 30 countable colonies were present.

### qRT-PCR

Overnight cultures in rich media were sub-cultured 1:50 in 5 mL and grown for 1 h. At that time, cultures were exposed to a stress of interest (vehicle, TPEN, HN_2_) and incubated at 37°C, 45°, 180 rpm for 60 min. At that time, cultures were spun down at 4,000 × *g* for 10 min and resuspended in 5 mL 1:1::acetone:ethanol at −20°C, which were then placed at −80°C overnight (*x* > 20 h). Pelleted samples were dried on ice, then suspended in 1 mL of Trizol (procured from Ambion), placed in Lysing Matrix B tubes, and lysed similarly to reported for Immunoblotting. Lysed samples were treated with chloroform, centrifuged, and the RNA-containing layer was collected for downstream preparation using a Qiagen RNAEasy Prep kit and subsequently TURBO DNA-free kit following manufacturer instructions. Samples were then subjected to Qiagen RNAEasy Prep kit steps for RNA Cleanup. RNA concentration and purity were quantified, and cDNA was synthesized using the iScript cDNA Synthesis Kit from Bio-Rad per manufacturer’s instructions. Measurement of relative expression level change was collected using the SYBR Green kit from Bio-Rad, optimized for a 12.5 µL total reaction volume, then analyzed using the calculation of 2^–∆∆Ct^.

### Catalase assays

Catalase activity was measured via qualitative and quantitative measures. Qualitative measurements involved preparing an overnight culture from a single colony of bacteria, which was allowed to grow in liquid tryptic soy broth for 24 h. Without otherwise being processed, 10 µL of overnight culture was dropped onto a plate containing TSA ± TPEN 10 µg/mL, and once dried, incubated statically at 37°C overnight. After 18 h of growth, bacteria were imaged using a Chemi-Doc. These plates were re-imaged after exposure to 20 µL of 20% H_2_O_2_, diluted from a 35% stock. Images were then evaluated for differences in gas production. Quantitative evaluation of catalase activity was done following Cayman Catalase assay kit instructions, visualized by readout of OD_520_ on an EPOCH spectrophotometer. Results were derived from OD_600_-normalized 10 µL of stationary liquid-phase culture grown for 20 h (49).

### Mouse infections

Four-week-old male and female C57BL6/J mice were ordered from Jackson Labs. In compliance with VUMC IACUC Protocol ID M1900043-01. Once 6 weeks of age, mice were subjected to anesthesia and retro-orbital systemic infection with *S. aureus* with or without *zigA*.Infection protocols follow those previously established (74, 83, 91–93). In compliance with the approved IACUC protocol, mice were weighed daily and euthanized if they met endpoints.At 4 days post-infection, all remaining mice were euthanized via CO_2_, where major organs were collected, homogenized via bead beating in NAVY bead lysis tubes (NextAdvance), serially diluted, and plated on TSA plates. Colonies were enumerated and recorded after 18 < *x* < 24 h of growth at 37°C.

### Antimicrobial sensitivity testing

Antimicrobial susceptibility testing was in part performed using the Kirby-Bauer disk diffusion method (94, 95). Testing was performed on 5% Mueller-Hinton agar using commercially available antimicrobial disks (BD) according to Clinical and Laboratory Standard Institute (CLSI) guidelines, M100-ed3037 (96). Additional testing, also in line with CLSI guidelines, VITEK 2 AST-GP75 Gram-Positive Susceptibility Card was used to evaluate the susceptibility of the tested *S. aureus* samples. E-Test Strip testing was completed for strains with and without the addition of TPEN to the Tryptic Soy Agar plate (97). Per standard procedures, after pouring and drying plates, an overnight culture of *S. aureus* or mutant was diluted to equivalent turbidity as a 0.5 MacFarland standard then aliquoted onto a plate of interest using a cotton swab. After liquid culture had dried, *E*-test strips were applied per manufacturer instructions, and plates were incubated statically at 37°C for 18–20 h (97), then imaged using a Bio-Rad Chemi-Doc using the “Stain-Free Blot” application.

### Data analysis

Data analysis made use of statistical approaches described in each figure caption. To conduct these analyses, this work made use of Excel version 2502, GraphPad version 10.4.2, Uniprot, a Bio-Rad Chemidoc MP Imaging System, Biognosys Spectronaut v.19.5.241,126, Scaffold v.5.3.3, ClustalO, iTOL, Inkscape, Jalview v.2.11.4.1, Canvas X version 20 build 914 (98–100).

## SUPPLEMENTAL MATERIAL

Supplemental figures spectrum.00454-26-s0001.docx Figures S1–S5.

ASM does not own the copyrights to Supplemental Material that may be linked to, or accessed through, an article. The authors have granted ASM a non-exclusive, world-wide license to publish the Supplemental Material files. Please contact the corresponding author directly for reuse.
