# Supplementary material for: Staphylococcus aureus ZigA is implicated in survival in zinc-deplete and genotoxic environments
Source: Microbiol Spectr. 2026 Jun 11;14(7):e00454-26. doi: 10.1128/spectrum.00454-26 (PMC13339975; doi:10.1128/spectrum.00454-26)
Supplement: Supplemental figures — Figures S1–S5. [file spectrum.00454-26-s0001.docx]

**
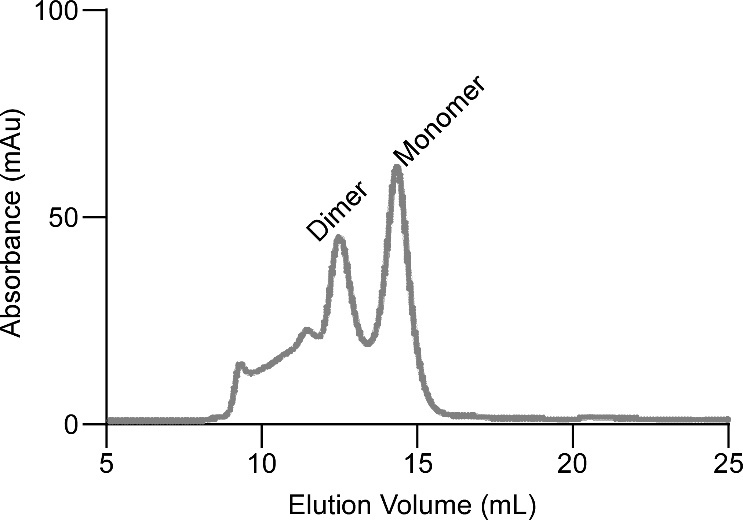
**

**Supplemental Figure 1: Size Exclusion Chromatography (SEC) showing recombinant *S. aureus* ZigA exists in an equilibrium between monomeric and dimeric states.** The SEC trace obtained at the final step of protein purification with no co-factors present shows two primary peaks eluting at volumes that suggest an equilibrium between monomeric and dimeric states.


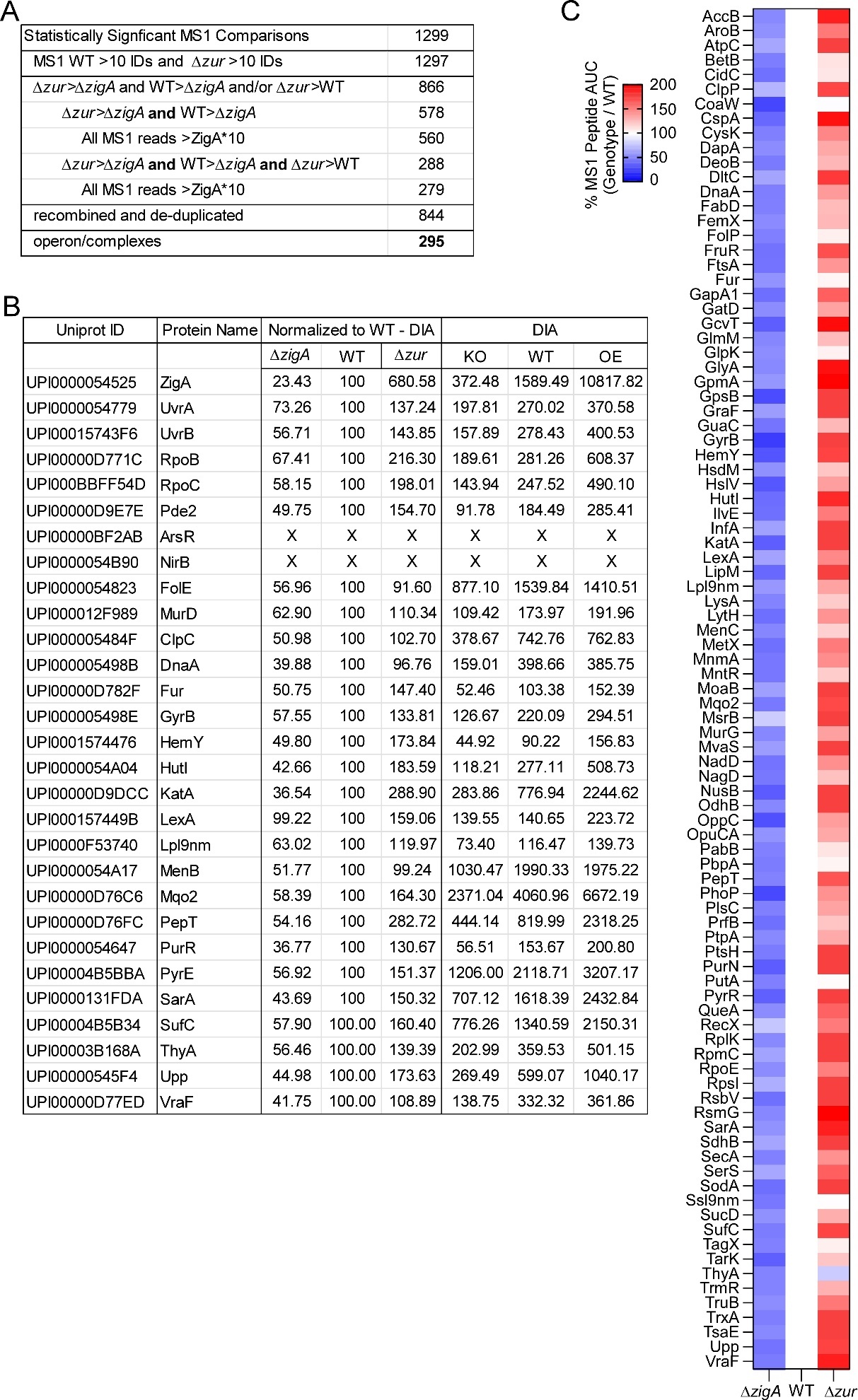


**Supplemental Figure 2: Co-immunoprecipitation of ZigA makes similar identifications by DDA and DIA analysis across multiple instruments. (A)** Process flow depicting the results of Co-IP MS1 analysis of DIA datasets obtained from a Bruker timsTOF instrument. **(B)** Listed details for data presented in Figure 4F, including Uniprot ID, Normalized (to WT) DIA, and average raw DIA timsTOF instruments. **(C)** Data associated with selected protein identities and normalized to their MS1 integrated area under the curve in WT strains detailed for statistically significant hits where multiple operon / protein complex members were classified as statistically significant hits.

**
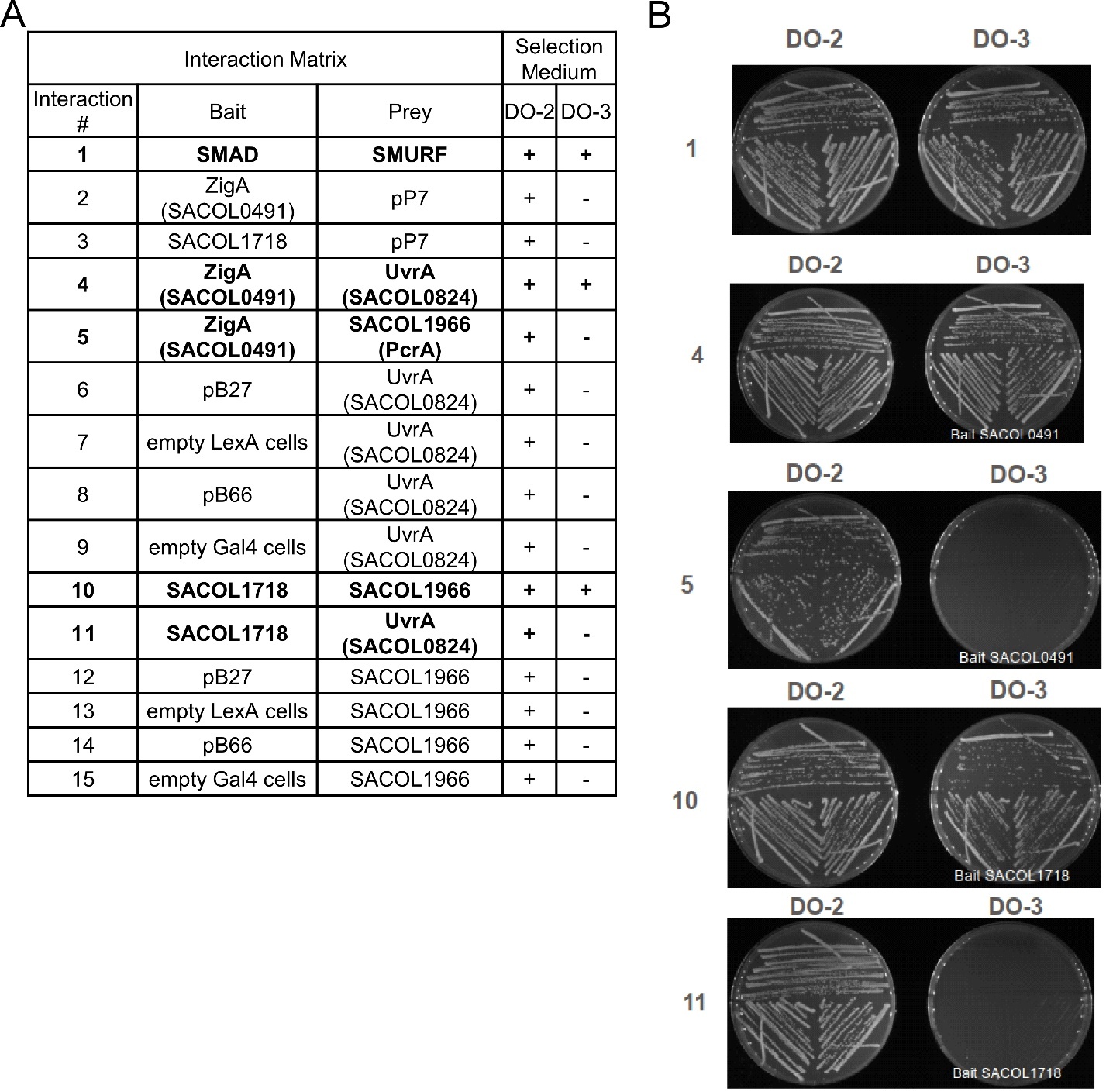
**

**Supplemental Figure 3: Yeast-2-Hybrid testing supports a robust interaction between UvrA (AA 665-866) and ZigA (AA 1-400). (A)** Listed relationships tested using *S. aureus* ZigA and fragments of *S. aureus* UvrA by Yeast-2-Hybrid, including standard controls and the results of interaction on DO-2 (non-selective) and DO-3 (selective media requiring interaction across protein fragments) media. **(B)** Visual depictions of specific relationships detailed in **(A)**, including plating results on DO-2 and DO-3 media.

**
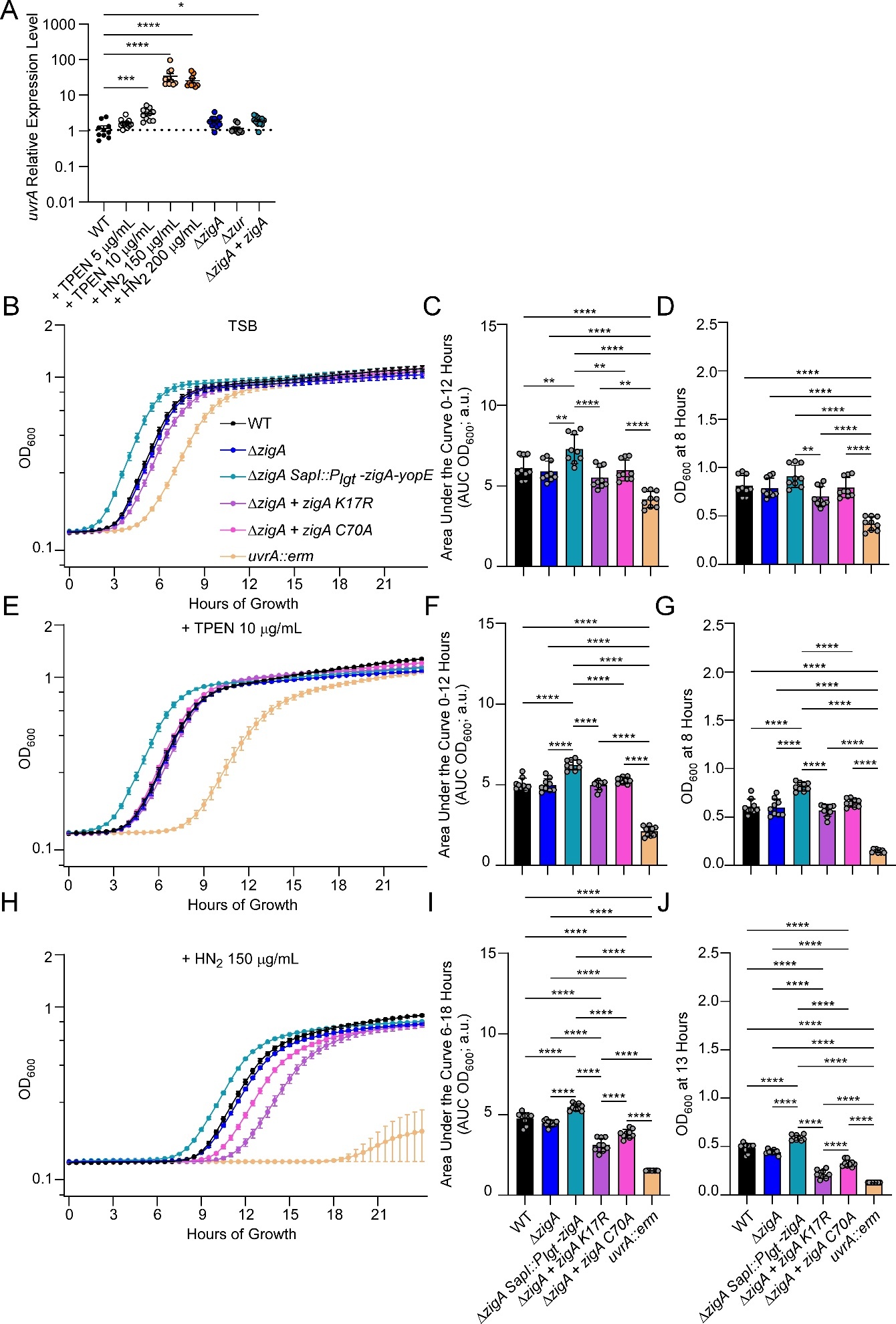
Supplemental Figure 4: Loss of *zigA* is associated with *S. aureus* growth defects requires both low Zn^2+^ and DNA damaging environments.** **(A)** Relative expression level (2^-∆∆Ct^) by SYBR Green based qRT-PCR of *uvrA* one hr after treatment with TSB + stressors. Statistics completed using Brown-Forsythe and Welch’s ANOVA tests with post-test *via* Dunnett’s T3 multiple comparison test, with individual variance computed for each comparison to NWMN alone. Data presented are the result of triplicate biological replicates and nine total replicates. **(B-J)** 24 hr growth assays of *S. aureus* with and without *zigA* and residues predicted to be relevant to GTPase activity (K17R) and Zn^2+^ binding (C70A), as well as a transposon mutant from the NTML library of *uvrA* in **(B-D)** TSB alone, **(E-G)** TPEN 7.5 µg/mL, and **(H-J)** HN_2_ 150 µg/mL. From the results defined in **(B, E, and H),** **(C, F, and I)** describe the area under the curve (AUC) in the 12 hrs surrounding the exponential phase of growth. In turn, **(D, G, and J)** describe the OD_600_ values of replicate curves at selected timepoints. All statistics **(B-J)** by ordinary one-way ANOVA with Tukey’s multiple comparison test, with a single pooled variance, all presented distributions are mean +/- standard deviation. * = p<0.05, ** = p<0.01, *** = p<0.005, **** = p<0.001. Sub-figures H and I do not include all comparisons to allow for effective visualization.

**
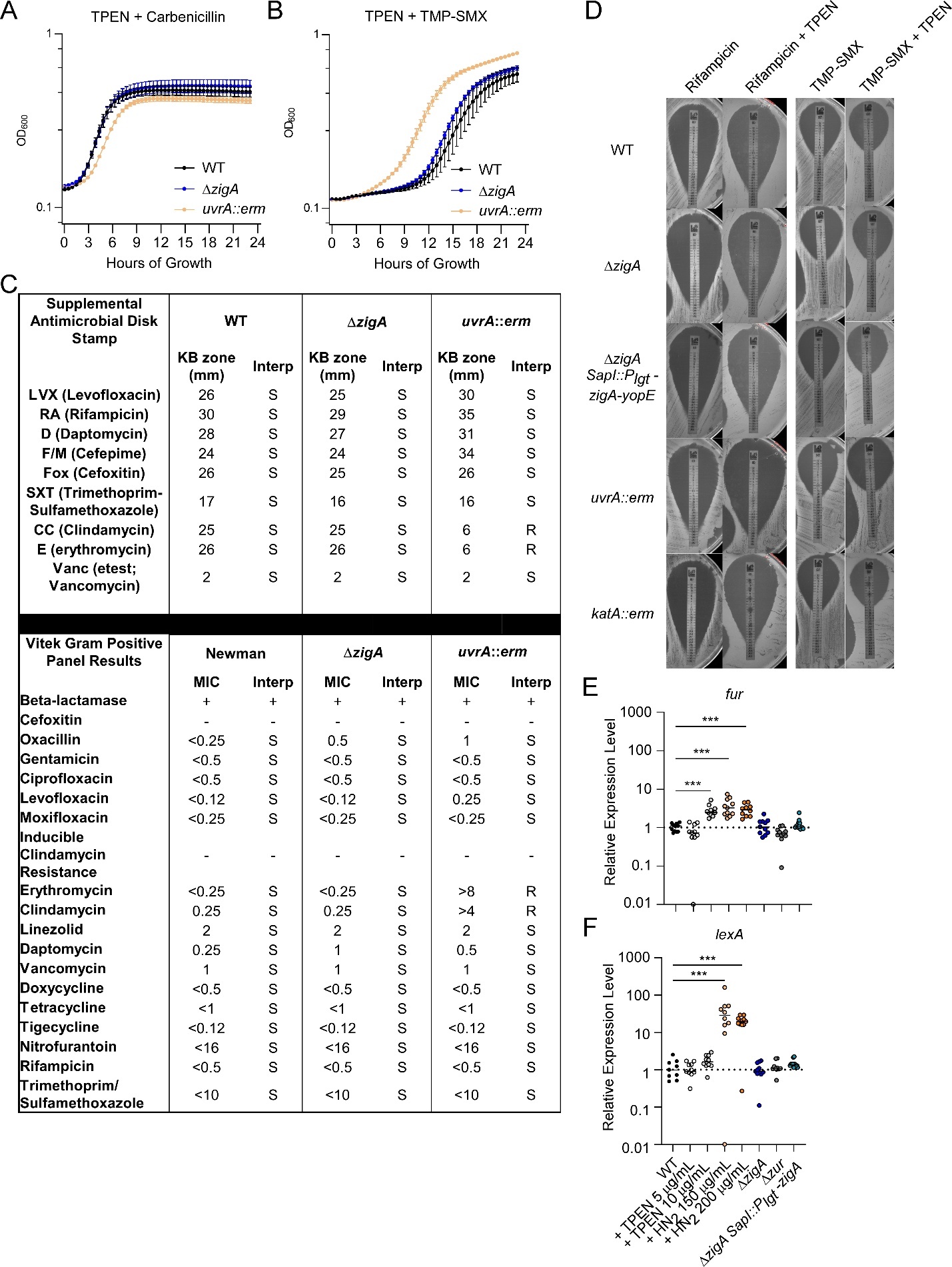
**

**Supplemental Figure 5**: **Antibiotic susceptibility of *S. aureus* does not significantly change in the absence of *zigA*.** **(A)** 24 hr growth assays of *S. aureus* treated with TPEN 5 μg/mL and Carbenicillin 0.625 μg/mL or **(B)** TPEN and trimethoprim-sulfamethoxazole 0.575 μg/mL. **(C)** Results of Vitek Gram Positive Panel and supplemental antimicrobial disk stamp assay for characterization of the antibiotic resistance of WT Newman, ∆*zigA,* or *uvrA*::*erm*. **(D)** E-test evaluation of antimicrobial susceptibility of strains with and without *zigA* relevant to potential non-UvrA clients (Rifampicin and RpoC, left; and trimethoprim-sulfamethoxazole and FolD, right). Relative expression level (2^-∆∆Ct^) by qRT-PCR of **(E)** *isdG* and **(F)** *lexA* one hr after treatment. Statistics using Kruskal-Wallis tests with post-hoc uncorrected Dunn’s test for each comparison to NWMN alone.
